# Management of Osilodrostat Therapy in Patients With Cushing's Syndrome: A Modified Delphi Consensus Panel

**DOI:** 10.1210/jendso/bvaf103

**Published:** 2025-06-27

**Authors:** Susan L Samson, Diane Donegan, Eliza B Geer, Murray B Gordon, Oksana Hamidi, Wenyu Huang, Adriana G Ioachimescu, Julie M Silverstein, Joanna L Spencer-Segal, Nicholas A Tritos, Kevin C J Yuen

**Affiliations:** Departments of Medicine and Neurologic Surgery, Mayo Clinic, Jacksonville, FL 32224, USA; Division of Endocrinology, Diabetes and Metabolism, Mayo Clinic, Rochester, MN 55905, USA; Departments of Medicine and Neurosurgery, Multidisciplinary Pituitary and Skull Base Tumor Center, Memorial Sloan Kettering Cancer Center, New York, NY 10021, USA; Division of Endocrinology, Allegheny Neuroendocrinology Center, Allegheny General Hospital, Pittsburgh, PA 15212, USA; Division of Endocrinology and Metabolism, University of Texas Southwestern Medical Center, Dallas, TX 75390, USA; Division of Endocrinology, Metabolism and Molecular Medicine, Northwestern University Feinberg School of Medicine, Chicago, IL 60611, USA; Departments of Medicine (Division of Endocrinology and Molecular Medicine) and Neurosurgery, Medical College of Wisconsin, Milwaukee, WI 53226, USA; Division of Endocrinology, Metabolism and Lipid Research, Washington University School of Medicine, St. Louis, MO 63110, USA; Department of Internal Medicine and Michigan Neuroscience Institute, University of Michigan, Ann Arbor, MI 48109, USA; Neuroendocrine Unit and Neuroendocrine and Pituitary Tumor Clinical Center, Massachusetts General Hospital and Harvard Medical School, Boston, MA 02114, USA; Barrow Pituitary Center, Barrow Neurological Institute and St. Joseph’s Hospital and Medical Center, University of Arizona College of Medicine and Creighton School of Medicine, Phoenix, AZ 85013, USA

**Keywords:** adrenal crisis, adrenal insufficiency, Cushing's disease, endogenous Cushing's syndrome, glucocorticoid withdrawal syndrome, osilodrostat

## Abstract

**Introduction:**

Endogenous Cushing's syndrome (CS) is a rare endocrine disorder that chronically exposes patients to supraphysiological cortisol levels. Primary therapy for CS consists of surgery. Medical therapies are also considered for many patients with CS, including those who are not surgical candidates or have persistent or recurrent hypercortisolism after surgery. Osilodrostat, an adrenal steroidogenesis inhibitor, demonstrated sustained efficacy and safety in phase 3 clinical trials and is currently approved to treat endogenous CS in Europe and the United States. Because of limited clinical experience, questions remain about how to individualize osilodrostat treatment for different clinical scenarios and special populations. Additional guidance from experts based on clinical study and real-world experiences with osilodrostat is needed.

**Methods:**

A modified Delphi consensus panel study was conducted consisting of 13 specialists from high-volume endocrinology centers with experience prescribing osilodrostat. Advisors participated in 3 consensus rounds (2 anonymous surveys, 1 virtual workshop) over approximately 10 months to provide guidance and recommendations on optimal osilodrostat use.

**Results:**

Over 2 surveys and a 2-hour virtual workshop, 26 statements related to osilodrostat achieved consensus among Delphi panelists and 5 were excluded. Topics included patient preparation before osilodrostat initiation, baseline testing, dosing at onset and during treatment, managing dose adjustments, monitoring during dose titration, and treatment alterations for planned and unexpected clinical events.

**Conclusion:**

Treatment guidance and recommendations for osilodrostat use were obtained using the Delphi method. These statements are intended to provide physicians with education and guidance on using osilodrostat to optimally treat patients with CS.

Endogenous Cushing's syndrome (CS) is a rare endocrine disorder caused by chronic excessive cortisol production, with an estimated prevalence ranging from 39 to 79 individuals per million depending on the population [1]. In ACTH-dependent CS, excessive ACTH is secreted by either a pituitary adenoma (Cushing's disease [CD]) or, less commonly, ectopically by tumors elsewhere in the body, many of which are neuroendocrine in origin [1]. In ACTH-independent CS, excess cortisol is produced by the adrenal glands in the context of an adrenal adenoma, adrenal cortical carcinoma, or macronodular adrenal hyperplasia.

Effective treatment of endogenous CS is crucial to avoid associated complications of excess cortisol exposure including metabolic, infectious, cardiovascular, musculoskeletal, neuropsychiatric, and hematologic sequelae. When possible, the primary treatment for endogenous CS is surgical removal of the culprit tumor. Although remission rates for CD can be 70% or higher at centers that perform transsphenoidal surgery in high numbers, surgery is not always curative or feasible [2, 3]. A systematic review of studies, each with ≥100 patients with CD, reported CD recurrence rates ranging from 1% to 26% for studies with <5 years of follow-up and 2% to 41% for >5 years of follow-up [4]. Other reviews have reported long-term recurrence rates as high as 66% [1, 2]. In 1 multicenter study, 50% of 220 patients did not achieve biochemical control after initial pituitary surgery [5]; in other studies, remission rates were significantly lower, and/or recurrence rates were significantly higher, after initial surgery in patients with a macroadenoma vs microadenoma (remission: 66–67% vs 89–91%, respectively; recurrence: 34–36% vs 11–12%, respectively) [6, 7]. Therefore, alternative treatment options are frequently necessary.

Medical therapy can be used to treat persistent or recurrent CS if surgery fails and is sometimes used in conjunction with radiation therapy. Other candidates for medical therapy include those who have biochemical evidence of CS but no identifiable tumor, those who have unresectable or metastatic ectopic neuroendocrine tumors or adrenal cancer, and those who are critically ill from CS. Medical therapy may also be needed preoperatively to address the effects of severe hypercortisolism and its associated complications prior to surgery; as an adjunctive “bridge therapy” while awaiting the clinical effect of radiation therapy; or at any time if surgery is either delayed, not feasible, or declined by the patient [1, 8].

Current medical therapies indicated for CS or CD and approved by the European Medicines Agency (EMA) and/or the US Food and Drug Administration (FDA) have been reviewed elsewhere [1, 9, 10]. These include adrenal steroidogenesis inhibitors (osilodrostat [FDA and EMA], levoketoconazole [FDA only] [11], ketoconazole [EMA only] [12], and metyrapone [EMA only] [13]), the somatostatin receptor ligand pasireotide (CD [FDA and EMA]) [14, 15], and the glucocorticoid (GC) receptor antagonist mifepristone (FDA only) [16, 17]. Other medical therapies that are used off-label in both Europe and the United States include the dopamine agonist cabergoline (CD) [18, 19], steroidogenesis inhibitors etomidate and mitotane [1], and combination therapies (eg, cabergoline as an add-on to ketoconazole, metyrapone, and/or pasireotide therapy) [18, 20, 21].

The adrenal steroidogenesis inhibitor osilodrostat was approved for CS in 2020 in Europe [22] and in 2025 in the United States, which expanded from the prior CD indication approved in 2020 (for adults who are not surgical candidates or whose surgery has not been curative) [23]. In phase 3 clinical trials (LINC 3 and LINC 4), osilodrostat demonstrated a rapid and sustained reduction in cortisol levels and durable, long-term (up to 204 weeks) efficacy in participants with persistent, recurrent, and newly diagnosed CD; further, improvements in clinical signs and symptoms, quality of life, and depression score outcomes were observed [24, 25]. In a pooled analysis of data from these phase 3 studies, normalization of late-night salivary cortisol (LNSC) and mean urinary free cortisol (UFC) levels led to improvements in outcomes up to week 72, indicating that treatment should aim for normalization of both parameters for optimal patient outcomes [26]. However, these clinical trials also reported substantial rates of symptoms of adrenal insufficiency (AI) and GC withdrawal syndrome (GWS), mainly during the dose titration phase [24, 27], that may negatively affect patient quality of life and treatment adherence [28, 29]. Results from the LINC 4 clinical study suggested that hypoadrenal adverse effects can be mitigated with slower titration [30], but there is still a need for additional guidance on how to utilize osilodrostat optimally during dose titration and when symptoms and unplanned events arise (eg, intercurrent illness).

To date, clinicians have accumulated over 4 years of experience using osilodrostat in clinical studies and real-world clinical settings. Collecting best practices from high-volume CS specialists can provide clinical guidance to improve confidence in how to initiate, titrate, and monitor patients with CS using osilodrostat. The Delphi process is a means of obtaining consensus from experts to coalesce informed, experience-based opinions on emerging technologies or therapies [31]. We used a modified Delphi process to obtain consensus on guiding statements related to osilodrostat initiation and management as an educational resource for endocrinologists and other clinicians who are considering utilizing this treatment to treat patients with CS.

## Methods

### Study Design

A modified Delphi process was used to obtain consensus from physician experts who have prescribed osilodrostat and/or served as investigators in clinical studies using osilodrostat for CS [32]. The consensus process was led by cochairs Dr. Susan L. Samson and Dr. Kevin C.J. Yuen. The process included 2 rounds of anonymized surveys followed by a 2-hour virtual workshop. This study was not prospectively registered.

The cochairs were responsible for panelist selection. A list of experienced physicians in the United States who practiced at high-volume endocrinology centers, prescribed osilodrostat, and/or participated in previous osilodrostat clinical studies were selected by the cochairs for consideration. Recruitment aimed for ≥10 Delphi panel advisors on the basis of panel sizes of Delphi studies for rare disease states and aimed at maximizing the diversity of clinical practices and levels of experience with osilodrostat [33, 34]. No more than 1 person representing 1 institution was invited at a time. Invitations were emailed to 17 individuals selected by the cochairs. In total, 5 declined, 1 did not respond, and 11 agreed to participate.

### Initial Statement Drafting

Cochairs drafted initial statements on the basis of their clinical experience and knowledge of the published literature. After virtual introductory meetings were held with advisors to explain the Delphi process, discuss objectives, and propose statement topics, the initial statements were emailed to the Delphi panel to solicit their feedback and additional statement suggestions. In accordance with feedback from the advisors, the decision was made to not include statements containing guidance already available in the osilodrostat package insert.

### Delphi Surveys

All statement surveys were built and distributed using the SurveyMonkey software platform and did not require a pilot survey. Delphi panel advisors (including the 2 cochairs) were sent an electronic link to the survey and were given approximately 2 weeks to complete each survey (Survey 1: November 30-December 15, 2023; Survey 2, July 2–16, 2024). Survey data output assigned a random respondent ID number to each advisor to preserve anonymity.

Advisors were asked to rate their level of agreement or disagreement with each statement and substatement using a 7-point Likert-type scale: 1 (“complete disagreement”), 2 (“disagreement”), 3 (“some disagreement”), 4 (“neither agreement nor disagreement”), 5 (“some agreement”), 6 (“agreement”), and 7 (“complete agreement”). One multiple-choice statement in Survey 1 (“How long before pituitary surgery should osilodrostat be interrupted?”) instead asked advisors to select from a list of options representing different numbers of days and was later revised to a Likert-scale statement for Survey 2. Each statement had a comment box for suggested statement modifications or additional input, and advisors were instructed to use the comment box to explain any score ≤2.

Statements that did not achieve consensus in survey 1 were revised by the cochairs on the basis of quantitative scores and qualitative advisor comments. The subsequent Survey 2 contained only the revised statements, not statements that had achieved consensus in Survey 1.

### Consensus Roundtable

After Survey 2 data were analyzed, a 2-hour virtual workshop was held on September 5, 2024, to discuss survey results, resolve statements that did not achieve consensus after Survey 2, and address remaining concerns about included statements. Minor word changes to final statements were permitted for the purpose of clarifying language but not to add new concepts to the statements.

### Data Analysis

Survey statement scores were descriptively analyzed after each Delphi round to determine whether consensus was achieved according to predetermined criteria. Consensus to include a statement was met if ≥80% of its responses had a score ≥6; to exclude a statement, ≥80% of its responses had to have a score ≤2. Response scores for a given statement were excluded from calculations if the responder both indicated a score of 4 (“neither agreement nor disagreement”) and indicated abstention or lack of experience in the statement comment box.

## Results

From 13 total Delphi panel advisors, 12 complete surveys and 1 incomplete survey were obtained for Survey 1; 11 complete surveys and 1 incomplete survey were obtained for Survey 2. Per request, Survey 2 was completed by an advisor 2 weeks after the survey end date. Consensus to include 16 out of 62 Survey 1 statements/substatements and 15 out of 43 Survey 2 statements/substatements was achieved based on predefined scoring criteria. No statements achieved consensus to exclude from either survey based on scoring. Survey 2 scores from 1 advisor were unblinded to the data analyst, but adding these scores did not change any consensus decisions and the panel remained blind to responder identities.

Twelve of the 13 advisors participated in the workshop and achieved consensus to include 9 additional statements/substatements. All advisors received a recording of the workshop and a comprehensive summary of statements achieving consensus. The advisor who was not able to attend the workshop endorsed outcomes via email after reviewing these materials.

**Figure 1.**
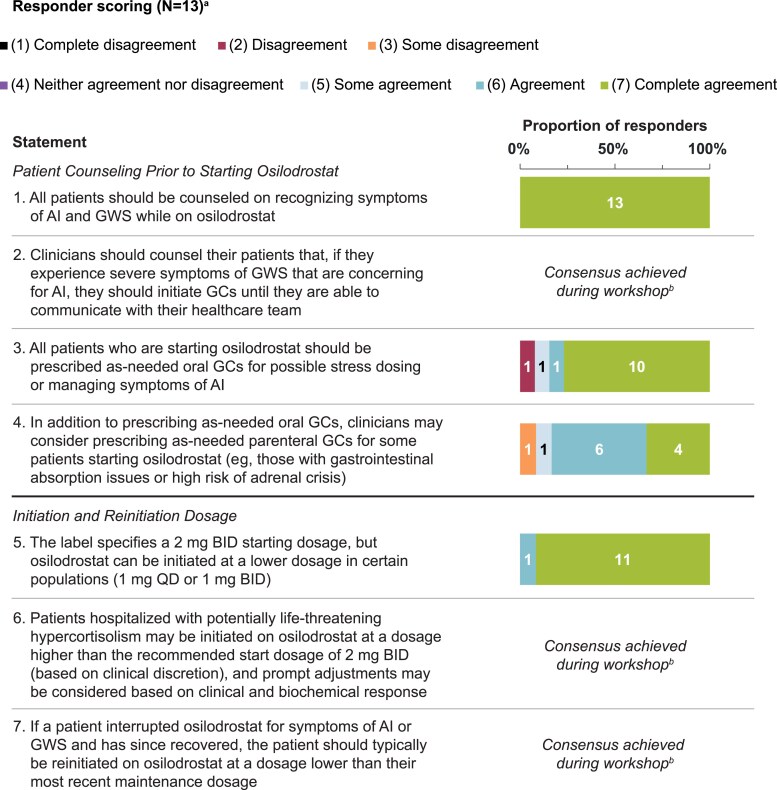
Delphi panel consensus on best practices for preparing patients to initiate osilodrostat and choose osilodrostat dosage at initiation and reinitiation. ^a^For each statement, n values within each bar indicate the number of respondents per score (11-13 total responders each). ^b^Consensus was achieved verbally during the workshop (n = 12) and endorsed in writing after the workshop (n = 1).Abbreviations: AI, adrenal insufficiency; BID, twice daily; GC, glucocorticoid; GWS, glucocorticoid withdrawal syndrome; QD, once daily.

**Figure 2.**
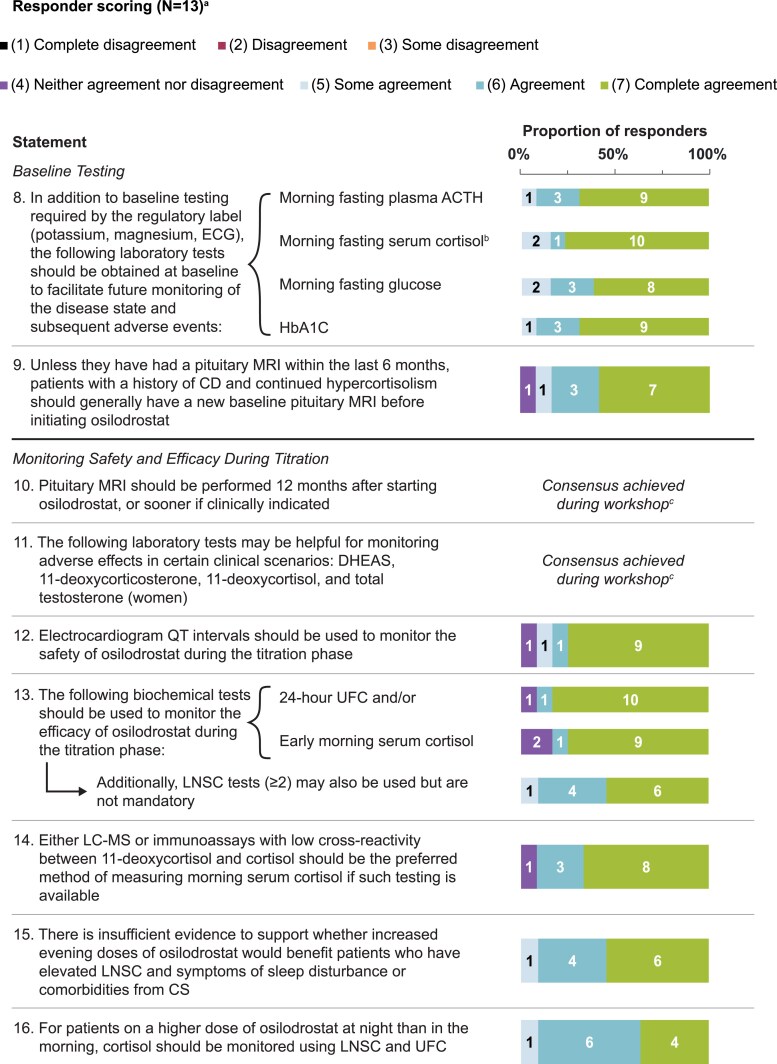
Delphi panel consensus on laboratory testing and imaging to acquire at baseline and throughout titration with osilodrostat. ^a^For each statement, n values within each bar indicate the number of respondents per score (11-13 total responders each). ^b^Nonmorning and nonfasted conditions are acceptable, with the caveat that food may increase cortisol levels. ^c^Consensus was achieved verbally during the workshop (n = 12) and endorsed in writing after the workshop by the final panel member.Abbreviations: BID, twice daily; CD, Cushing's disease; CS, Cushing's syndrome; DHEAS, dehydroepiandrosterone sulfate; ECG, electrocardiogram; HbA1c, glycated hemoglobin; LC-MS, liquid chromatography–mass spectrometry; LNSC, late-night salivary cortisol; MRI, magnetic resonance imaging; UFC, urinary free cortisol.

**Figure 3.**
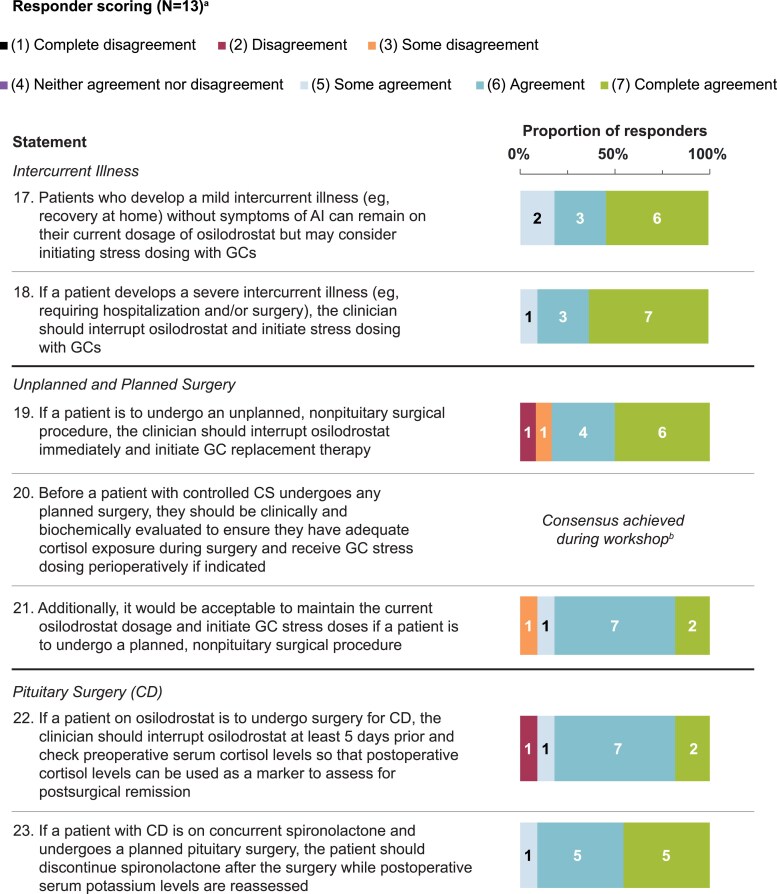
Delphi panel consensus on how to modify treatment with osilodrostat and concurrent medications in the event of intercurrent illness, planned surgeries, and unplanned surgeries. ^a^For each statement, n values within each bar indicate the number of respondents per score (11-13 total responders each). ^b^Consensus was achieved verbally during the workshop (n = 12) and endorsed in writing after the workshop by the final panel member. Abbreviations: AI, adrenal insufficiency; CD, Cushing's disease; CS, Cushing's syndrome; GC, glucocorticoid.

**Figure 4.**
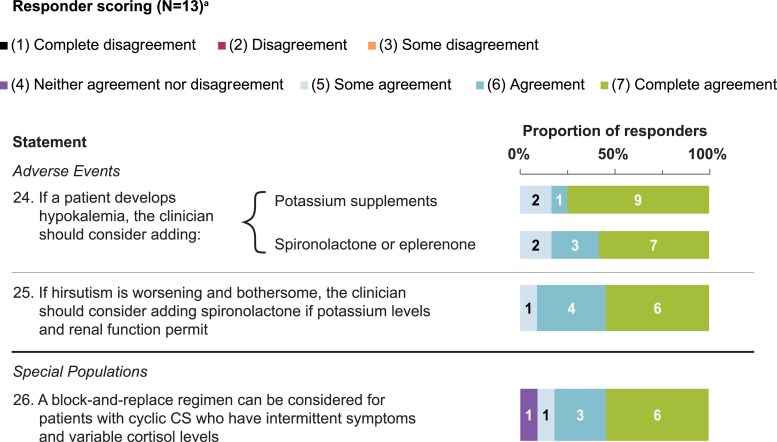
Delphi panel consensus on treatment modifications for adverse events and in special populations. ^a^For each statement, n values within each bar indicate the number of respondents per score (11-13 total responders each). Abbreviation: CS, Cushing's syndrome.

Statements that achieved consensus are in Table 1, with scoring in Figs. 1 to 4. During initial statement drafting, the panel decided to mostly exclude guidance that was redundant with the product label to focus on guidance beyond what is recommended by the label [22, 23]. Five statements were excluded regardless of consensus scoring outcome, either because they were considered out of scope (eg, redundant with the product label) or because they were addressed by other revised statements (Table 2).

**Table 1.** Delphi panel consensus statements

**Patient counseling prior to starting osilodrostat**
1. All patients should be counseled on recognizing symptoms of AI and GWS while on osilodrostat.
2. Clinicians should counsel their patients that, if they experience severe symptoms of GWS that are concerning for AI, they should initiate GCs until they are able to communicate with their healthcare team.
3. All patients who are starting osilodrostat should be prescribed as-needed oral GCs for possible stress dosing or managing symptoms of AI.
4. In addition to prescribing as-needed oral GCs, clinicians may consider prescribing as-needed parenteral GCs for some patients starting osilodrostat (eg, those with gastrointestinal absorption issues or high risk of adrenal crisis).
**Initiation and reinitiation dosage**
5. The label specifies a 2 mg BID starting dosage, but osilodrostat can be initiated at a lower dosage in certain populations (1 mg QD or 1 mg BID).
6. Patients hospitalized with potentially life-threatening hypercortisolism may be initiated on osilodrostat at a dosage higher than the recommended start dosage of 2 mg BID (based on clinical discretion), and prompt adjustments may be considered based on clinical and biochemical response.
7. If a patient interrupted osilodrostat for symptoms of AI or GWS and has since recovered, the patient should typically be reinitiated on osilodrostat at a dosage lower than their most recent maintenance dosage.
**Baseline testing**
8. In addition to baseline testing required by the regulatory label (potassium, magnesium, ECG), the following laboratory tests should be obtained at baseline to facilitate future monitoring of the disease state and subsequent adverse events: Morning fasting plasma ACTH Morning fasting serum cortisol*^a^* Morning fasting glucose HbA1c
9. Unless they have had a pituitary MRI within the last 6 months, patients with a history of CD and continued hypercortisolism should generally have a new baseline pituitary MRI before initiating osilodrostat.
**Monitoring safety and efficacy during titration**
10. In patients with CD, pituitary MRI should be performed 12 months after starting osilodrostat or sooner if clinically indicated.
11. The following laboratory tests may be helpful for monitoring adverse effects in certain clinical scenarios: DHEAS 11-deoxycorticosterone 11-deoxycortisol Total testosterone (women)
12. Electrocardiogram QT intervals should be used to monitor the safety of osilodrostat during the titration phase.
13. The following biochemical tests should be used to monitor the efficacy of osilodrostat during the titration phase: 24-hour UFC and/or Early morning serum cortisol Additionally, LNSC tests (≥2) may also be used but are not mandatory.
14. Either LC-MS or immunoassays with low cross-reactivity between 11-deoxycortisol and cortisol should be the preferred method of measuring morning serum cortisol if such testing is available.
15. There is insufficient evidence to support whether increased evening doses of osilodrostat would benefit patients who have elevated LNSC and symptoms of sleep disturbance or comorbidities from CS.
16. For patients on a higher dose of osilodrostat at night than in the morning, cortisol should be monitored using LNSC and UFC.
**Intercurrent illness**
17. Patients who develop a mild intercurrent illness (eg, recovery at home) without symptoms of AI can remain on their current dosage of osilodrostat but may consider initiating stress dosing with GCs.
18. If a patient develops a severe intercurrent illness (eg, requiring hospitalization and/or surgery), the clinician should interrupt osilodrostat and initiate stress dosing with GCs.
**Unplanned and planned surgery**
19. If a patient is to undergo an unplanned, nonpituitary surgical procedure, the clinician should interrupt osilodrostat immediately and initiate GC replacement therapy.
20. Before a patient with controlled CS undergoes any planned surgery, they should be clinically and biochemically evaluated to ensure they have adequate cortisol exposure during surgery and receive GC stress dosing perioperatively if indicated.
21. Additionally, it would be acceptable to maintain the current osilodrostat dosage and initiate GC stress doses if a patient is to undergo a planned, nonpituitary surgical procedure.
**Pituitary surgery (CD)**
22. If a patient on osilodrostat is to undergo surgery for CD, the clinician should interrupt osilodrostat at least 5 days prior and check preoperative serum cortisol levels so that postoperative cortisol levels can be used as a marker to assess for postsurgical remission.
23. If a patient with CD is on concurrent spironolactone and undergoes a planned pituitary surgery, the patient should discontinue spironolactone after the surgery while postoperative serum potassium levels are reassessed.
**Adverse events**
24. If a patient develops hypokalemia, the clinician should consider adding: Potassium supplements Spironolactone or eplerenone
25. If hirsutism is worsening and bothersome, the clinician should consider adding spironolactone if potassium levels and renal function permit.
**Special populations**
26. A block-and-replace regimen can be considered for patients with cyclic CS who have intermittent symptoms and variable cortisol levels.

Abbreviations: AI, adrenal insufficiency; BID, twice daily; CD, Cushing's disease; CS, Cushing's syndrome; DHEAS, dehydroepiandrosterone sulfate; ECG, electrocardiogram; GC, glucocorticoid; GWS, glucocorticoid withdrawal syndrome; HbA1c, glycated hemoglobin; LC-MS, liquid chromatography–mass spectrometry; LNSC, late-night salivary cortisol; MRI, magnetic resonance imaging; QD, once daily; UFC, urinary free cortisol.

^a^Nonmorning and nonfasted conditions are acceptable, with the caveat that food may increase cortisol levels.

**Table 2.** Statements not included for reasons unrelated to consensus survey scoring

Delphi stage	Statement/Substatements removed	Reason for removal
Survey 2	Baseline hypercortisolism can be measured using the following tests: 24-hour UFC LNSC (single) LNSC (multiple) Dexamethasone suppression test	Revised to consolidate the substatements that achieved consensus in Survey 1 into a new, single statement (next row)
Workshop	For the purpose of monitoring future osilodrostat need in a patient with persistent CD after a failed surgery or recurrence after surgical remission, baseline hypercortisolism can be determined using 24-hour UFC, LNSC, and/or dexamethasone suppression tests	Considered out of scope Not specific to osilodrostat use
Workshop	The following laboratory tests obtained under fasted morning conditions are informative, but not mandatory, for monitoring for future disease state and adverse events: Magnesium	Considered product label guidance
Survey 2	The following parameters should be used to monitor the safety of osilodrostat during the titration phase: Potassium Magnesium	Considered product label guidance
Survey 1	For a patient undergoing a planned, nonpituitary surgical procedure, osilodrostat can be interrupted or reduced on the day of surgery	The subject of this statement was combined with another revised statement*^a^*

Abbreviations: CD, Cushing's disease; LNSC, late-night salivary cortisol; UFC, urinary free cortisol.

^a^See statement 19 in Fig. 3.

### Patient Counseling Prior to Starting Osilodrostat

Patients receiving medical therapy for CS are at risk of AI, which can develop into life-threatening adrenal crisis if left untreated, at any time during osilodrostat treatment, and especially during events in which hypothalamic–pituitary–adrenal axis activation is normally expected [35]. Recent case reports have also documented 4 patients with CD who developed AI while on osilodrostat therapy that was prolonged after therapy discontinuation [36-38]. The panel unanimously agreed that patients should be counseled on how to recognize and differentiate symptoms of AI and GWS before initiating osilodrostat (Table 1 and Fig. 1, statement 1). Patients should also be educated that GWS symptoms are common to some extent and can last for weeks to several months after treatment initiation [29]. Symptoms of GWS include fatigue, myalgias, anorexia, and nausea, and findings indicative of AI include extreme fatigue (asthenia) and lethargy, vomiting, presyncope, dizziness, hypoglycemia, hypotension, hyponatremia, postural hypotension/presyncope or syncope, and concordant low morning cortisol levels [29]. Many symptoms overlap between AI and GWS, making it difficult to distinguish clinically which is being experienced without confirmatory biochemical tests [29, 39]. Other conditions can also impact AI and GWS symptoms, such as replacement therapies for endocrine deficiencies, including hypothyroidism, and the unmasking of inflammatory or autoimmune disorders by lowering cortisol levels [40].

The panel unanimously agreed that patients should be counseled to take GCs for severe GWS or AI symptoms until they can communicate with their clinician (Table 1 and Fig. 1, statement 2). Similar to guideline recommendations for primary or secondary AI treatment, hydrocortisone would be the usual choice as the most physiologic GC [41, 42]. Severe symptoms that would prompt empiric use of the prescribed GCs include those that are potentially indicative of AI (described in the previous paragraph). Importantly, symptoms of AI may not be worse than those of GWS. As it can be difficult to discern GWS from AI symptoms, statement 2 is primarily intended to prevent possible adrenal crisis in times of uncertainty until patients can reach their clinician. Some advisors preferred to counsel patients experiencing severe symptoms to temporarily interrupt osilodrostat while taking GCs and resume osilodrostat using a lower dose at a later date. However, other advisors preferred that patients be counseled to not interrupt osilodrostat unless they have been instructed by their prescriber. Overall, the overlap in GWS and AI symptoms and the potential influence of other confounding conditions emphasizes the importance of educating patients to seek clinical attention if they experience new or severe symptoms suggestive of AI after initiating osilodrostat.

The consensus was to prescribe oral GCs when starting osilodrostat for patients to take as needed for hypocortisolemic symptoms and to consider also prescribing as-needed parenteral GCs for individuals who may be unable to receive oral therapies because of, for example, vomiting (Table 1 and Fig. 1, statements 3 and 4), consistent with other recommendations and with clinical practice for patients with AI [43]. One advisor disagreed with prescribing oral GCs routinely, as they could be used inappropriately, while others were concerned that prescribing parenteral GCs at treatment start could delay treatment or overwhelm patients with information. As an alternative, patients could be educated about and prescribed parenteral GCs soon after osilodrostat initiation.

### Osilodrostat Initiation and Reinitiation Dosage

Advisors agreed that osilodrostat can be initiated at a dosage other than the label-recommended 2 mg twice-daily (BID) dosage in certain clinical scenarios (Table 1 and Fig. 1, statements 5 and 6) [22, 23]. A lower starting dosage could minimize GWS and AI symptoms and may be appropriate for patients with mild to moderate hypercortisolism. Additionally, those with liver dysfunction, concomitant use of a strong CYP3A4 inhibitor, or of Asian race may require a lower initiation dosage because of observed increases in osilodrostat exposure and bioavailability in these populations [22, 23, 44]. On the other hand, CYP3A4 and CYP2B6 inducers may reduce osilodrostat efficacy and necessitate a higher osilodrostat dosage, as would discontinuing a strong CYP3A4 inhibitor [23]. Information on CYP3A4 and CYP2B6 inducers and inhibitors, including their interactions with osilodrostat and recommended dosage modifications, has been previously summarized [35]. Furthermore, a higher initiation dosage may be appropriate for individuals with life-threatening hypercortisolism. A higher osilodrostat dosage (eg, 10-30 mg BID) with GC replacement has been implemented for a “block-and-replace” approach for patients hospitalized with ectopic CS and/or UFC >10 × the upper limit of normal (ULN) to rapidly decrease cortisol levels, minimize AI risk by preemptively replacing with GCs, and allow other treatments to start immediately (eg, anticancer treatment for ectopic CS or adrenocortical carcinoma) [35, 45, 46]. One advisor cautioned that higher doses may not be the best option for patients who are clinically unstable; however, osilodrostat monotherapy has been initiated successfully at initiation dosages higher than the label recommendation, both in patients with serum cortisol levels >145 mcg/dL (>4000 nmol/L) (osilodrostat 20 mg/day) and in patients with 24-hour UFC >10 × ULN (osilodrostat up to 40 mg/day) [45, 46].

For patients who have interrupted osilodrostat for symptoms of AI or GWS but have since recovered, the consensus was that osilodrostat should typically be reinitiated at a dosage lower than the most recent maintenance dosage (Table 1 and Fig. 1, statement 7). Resuming osilodrostat at a lower dosage could be appropriate if AI was biochemically verified with low morning serum cortisol, but resuming at the most recent maintenance dose could be acceptable if interruption due to AI was precipitated by an intercurrent illness (eg, gastroenteritis) or if the patient was already on a low dose (eg, 1 mg daily or BID). In cases of biochemically confirmed AI and/or adrenal crisis, it may be prudent to discuss recommendations for GC stress dosing with the patient for future intercurrent illness. To avoid inadvertently restarting osilodrostat during ongoing AI, which can potentially persist for months after discontinuing osilodrostat [36-38], it would be reasonable to test patients with a history of osilodrostat use for adrenal function recovery or for AI if symptoms potentially indicative of AI develop. Persistent primary AI can be verified with morning ACTH and cortisol after osilodrostat discontinuation to rule out alternate AI etiologies.

### Baseline Testing and Monitoring Titration

Statements related to laboratory testing and medical imaging before and during osilodrostat treatment are shown in Table 1 and Fig. 2 (statements 8-16). There was strong agreement to obtain early morning fasted serum cortisol, glucose, and plasma glycated hemoglobin and ACTH levels at baseline, to monitor for future disease state progression and adverse events. Importantly, as indicated in the product label, magnesium and potassium levels should be measured at baseline so that correction of hypomagnesemia and hypokalemia can be performed before osilodrostat initiation [22 , 23]. An advisor additionally recommended that magnesium and potassium levels be closely monitored during osilodrostat dose changes.

Glucose and glycated hemoglobin levels are obtained to assess for glucose intolerance or overt diabetes, monitor for glucose tolerance improvements when cortisol levels are lowered during osilodrostat treatment [24, 25], and inform whether any concurrent antihyperglycemia therapy requires modification. Likewise, other surrogate measures of hypercortisolism (eg, blood pressure, serum lipids, bone mineral density) can be obtained to monitor for other improvements to other hypercortisolemic comorbidities (eg, hypertension, hyperlipidemia) and determine whether corresponding treatments require modification [29, 47].

Patients may experience increases from baseline in ACTH levels; of note, those with adrenal CS will likely have low plasma ACTH levels at baseline that can rise during treatment. In the phase 3 LINC3 study, ACTH levels increased and did not stabilize until ≥72 weeks after osilodrostat initiation [25]. While some degree of ACTH rise can be expected after osilodrostat initiation, substantial and/or rapid ACTH increases can be observed with the rapid growth of an ACTH-producing tumor, such as with Nelson's syndrome [48]. However, no cutoff value for ACTH has been determined that accurately predicts pituitary tumor progression; thus, ACTH may be an unreliable predictor of tumor growth in patients with CD, especially if levels have not yet stabilized after the expected increase following osilodrostat initiation [24, 48]. Finally, advisors recommended that 24-hour UFC and LNSC also be measured at baseline to quantify the degree of hypercortisolism and for osilodrostat treatment monitoring.

#### Imaging

Patients with CD should have a new baseline pituitary magnetic resonance imaging (MRI) scan before initiating osilodrostat, unless one was obtained within the last 6 months of treatment initiation (Table 1 and Fig. 2, statement 9). However, clinicians should consider factors such as whether the tumor is a macroadenoma vs microadenoma, whether significant tumoral tissue remains postoperatively, and whether the individual received medical therapy since their last MRI scan. For example, performing a baseline MRI before treatment initiation would be reasonable even if a recent (<6 months) MRI scan is available for cases in which a substantial tumor remnant was noted at the last MRI or if the patient received medical therapy, as all medical therapies (apart from pasireotide or cabergoline) could theoretically induce tumor enlargement due to the removal of feedback inhibition from hypercortisolemia.

The panel agreed that a follow-up pituitary MRI should be generally performed approximately 12 months after starting osilodrostat (Table 1 and Fig. 2, statement 10) to monitor for tumor growth [10]. However, pituitary imaging could be obtained sooner depending on the baseline tumor size, occurrence of symptoms suggestive of mass effect (eg, new or worsening headache, visual changes, ophthalmoplegia), substantial increase in ACTH, or worsening of hypercortisolism, based on clinical judgement. The LINC3 core study phase reported 4 (3%) osilodrostat discontinuations due to increased tumor volume [27], and 4 additional core study discontinuations (8 in total) were due to events possibly related to tumor enlargement and associated sequelae, including diplopia, cranial nerve 6 paralysis, and tumor invasion [25]. The core study phase reported an equivalence between the proportions of patients with either a ≥20% increase or ≥20% decrease in tumor volume (week 24, 29% and 30%, respectively; week 48, 33% and 38%, respectively) [27], and in the extension phase, these proportions were 39% and 30%, respectively, at week 72 [25].

#### Optional laboratory testing

Other adrenal products including the cortisol precursor 11-deoxycortisol, aldosterone precursor 11-deoxycorticosterone, adrenal androgen dehydroepiandrosterone sulfate (DHEAS), and total testosterone (females) were considered useful tests to monitor for adverse events in specific scenarios (Table 1 and Fig. 2, statement 11). For example, checking 11-deoxycorticosterone may be helpful to confirm whether worsening hypertension or hypokalemia is due to osilodrostat. In addition, checking testosterone and DHEAS may help to confirm biochemical hyperandrogenism for females who develop worsening hirsutism or acne, which can help to guide management (eg, treatment with spironolactone). Whether these tests are needed may also depend on which tests were abnormal before treatment and the underlying cause of CS. In the LINC3 study, mean DHEAS levels in females decreased during the study, while testosterone increased initially and then stabilized [27]. One advisor recommended that measuring testosterone levels in females should only be symptom-driven and that free testosterone levels (ideally by equilibrium dialysis) may be more reliable than total testosterone assays in CS patients because of lower SHBG associated with CS.

#### Safety and efficacy monitoring

Advisors recommended using electrocardiogram (ECG) QT intervals to monitor safety during treatment titration (Table 1 and Fig. 2, statement 12), in alignment with product label recommendations [22, 23]. Furthermore, clinicians should assess the cardiac history of the patient and whether they are using other drugs with the potential to prolong the QT interval while using osilodrostat to determine whether more frequent ECG monitoring is needed. Additional ECGs could be considered at dosage increases.

Efficacy should be monitored during osilodrostat dose titration using 24-hour UFC and early morning serum cortisol tests (Table 1 and Fig. 2, statement 13); additionally, multiple LNSC tests could be used [10]. Morning serum cortisol measurements can be used to evaluate for AI development in conjunction with clinical symptoms and signs, to monitor patients with significantly elevated serum cortisol levels, and to assess efficacy for planned block-and-replace therapy. Additionally, because serum cortisol results are often available within a day, they can be useful while waiting for LNSC and UFC results that can take a week or more. Patients with baseline 24-hour UFC in the normal range but with abnormal LNSC could benefit from continued LNSC monitoring for efficacy and titration; in a recent study, normalization of both UFC and LNSC led to greater reductions in blood pressure and fasting plasma glucose compared with UFC normalization alone [26]. Clinicians should be aware that serum cortisol measurements in women taking oral contraceptive pills may be misleading, as oral estrogen can increase total serum cortisol levels by increasing levels of cortisol-binding globulin [49]. Of note, it is important to consider that oral estrogen can exacerbate the hypercoagulability of CS, and this should be taken into account if oral estrogen-containing contraceptives are continued [10]. Additionally, advisors cautioned that assessing efficacy with frequent UFC collections can become a burden for the patient.

For accuracy and chemical specificity, liquid chromatography–mass spectrometry (LC-MS) should be the preferred method of measuring cortisol, when available (Table 1 and Fig. 2, statement 14) [27, 50-53]. However, medical facilities without in-house access to LC-MS could use immunoassays with low cross-reactivity between 11-deoxycortisol and cortisol and reserve sending samples out to external LC-MS services for situations in which cross-reactivity is suspected.

#### Asymmetric dosing strategy

Asymmetric dosing of osilodrostat has been considered because the circadian and ultradian rhythm of adrenal cortisol secretion is often dysregulated in CS [54]. Using a higher dose of osilodrostat in the evening relative to the morning dose is intended to mimic lower nocturnal physiological cortisol levels and achieve nadir cortisol levels in early morning hours, based on a metyrapone study in patients with adrenal nodules with mild autonomous cortisol secretion (MACS). Patients with MACS vs those without MACS had elevated nighttime (6 Pm to 2 Am) cortisol levels that normalized when metyrapone was administered at 6 Pm (500 mg) and 10 Pm (250 mg); although there was speculation that this could alleviate some symptoms and comorbidities of MACS, such outcomes were not reported and sleep quality was not measured [55]. Advisors agreed that there was insufficient evidence in patients with CS to support whether using a higher evening dose of osilodrostat would benefit patients with elevated LNSC and symptoms of sleep disturbance and/or CS comorbidities (Table 1 and Fig. 2, statement 15). Higher LNSC levels predicted worse sleep outcomes in a study of generally healthy participants at the within-person level, suggestive of a possible link between elevated LNSC and sleep disturbance [56]. To date, asymmetric dosing has only shown benefits for patients with CS in anecdotal cases, such as a case study in which improved sleeping pattern and LNSC normalization were achieved in a patient with mild CD after switch from a 1 mg BID dosage to 1 mg in the morning and 2 mg at night [35].

If patients are on a higher dose of osilodrostat at night compared with the morning, they should have cortisol measured using both LNSC and UFC (Table 1 and Fig. 2, statement 16). With asymmetric dosing, a higher LNSC result could indicate an inadequate evening osilodrostat dose. One advisor recommended that patients on an asymmetric dosing strategy utilize LNSC to guide dose adjustments of osilodrostat in the evening, despite normal UFC and/or morning serum cortisol levels. However, as there is still a lack of data for asymmetric dosing strategies overall, the utility and benefits of administering osilodrostat in either the evening or at bedtime requires further research.

### Osilodrostat Modification for Illness and Nonpituitary Surgery

Several clinical scenarios were discussed to determine whether interrupting, reducing, and/or maintaining osilodrostat dosage would be appropriate responses. For patients who develop a minor intercurrent illness without symptoms of AI, the panel agreed that patients can maintain current osilodrostat dosing and potentially initiate GC stress dosing (Table 1 and Fig. 3, statement 17). One advisor recommended that patients should be educated on short-term GC stress dose use during these “sick days” of acute intercurrent illness, preferably in writing. For example, patients could be instructed to initiate GCs at 2 to 3 times the physiologic dose (“sick dose”) for 2 to 3 days if febrile or gastrointestinal symptoms develop as part of the illness. Gastrointestinal symptoms such as vomiting and diarrhea may require a patient to receive sick doses parenterally. Further, since these symptoms can also overlap with AI symptoms, 1 advisor recommended interrupting osilodrostat and initiating sick dose GCs until either the gastrointestinal illness resolves or the cause is determined and treated.

The recommended course of action for patients who experience severe illness that requires hospitalization and/or surgery, or who are undergoing any unplanned (urgent or emergent), nonpituitary surgery, was to interrupt osilodrostat treatment and initiate GC stress dosing (Table 1 and Fig. 3, statements 18 and 19). Advisors agreed that patients should be treated with stress GCs in this scenario regardless of whether osilodrostat treatment is interrupted because the timeline of cortisol increases after osilodrostat discontinuation may be hard to predict.

**Figure 5.**
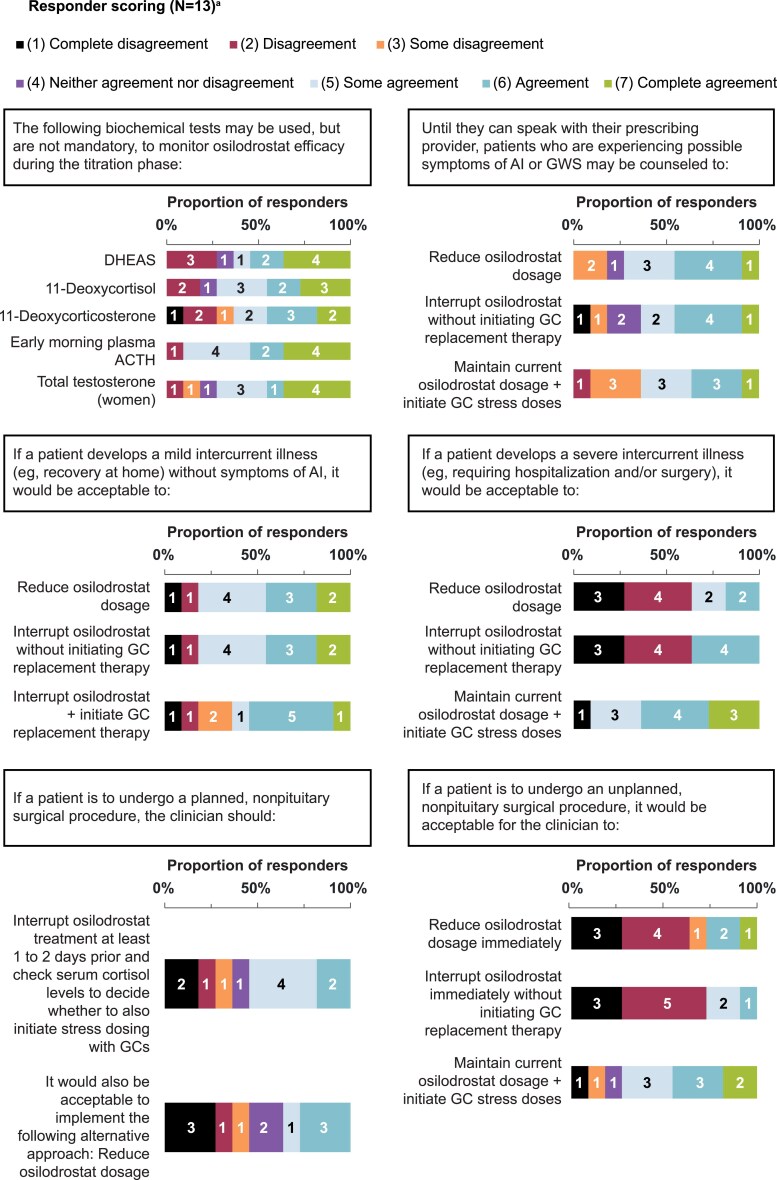
Delphi panel statements and substatements that did not achieve consensus during the virtual workshop. Survey 2 scores are shown with representative feedback comments. ^a^For each statement, n values within each bar indicate the number of respondents per score (11-13 total responders each). Abbreviations: AI, adrenal insufficiency; DHEAS, dehydroepiandrosterone sulfate; GC, glucocorticoid; GWS, glucocorticoid withdrawal syndrome; UFC, urinary free cortisol.

Similarly, perioperative GC stress dosing should be used as indicated for patients with controlled CS undergoing any planned surgery (Table 1 and Fig. 3, statement 20). Advisors also agreed that patients undergoing an elective, nonpituitary surgery could maintain their osilodrostat dosage and initiate stress GCs (Table 1 and Fig. 3, statement 21) as an alternative to reducing osilodrostat dosage or interrupting treatment 1 to 2 days ahead of the planned surgery (Fig. 5). Importantly, reducing osilodrostat dosage without initiating stress GCs was not advised as it would not guarantee adequate hypothalamic–pituitary–adrenal axis response to stress. One advisor preferred to interrupt osilodrostat treatment for all surgeries while another would only interrupt treatment for major procedures, as some patients may not need perioperative GC replacement with preoperative interruption of osilodrostat. Some advisors found it acceptable to interrupt osilodrostat at least a week ahead of a planned surgery; however, residual drug effects from osilodrostat may last longer than 7 days after interruption, with some reports of patients having prolonged hypocortisolemia after discontinuation [36-38]. Conversely, interrupting too far in advance (vs the day of the procedure) could lead to recurrent hypercortisolism that potentially could adversely affect surgery outcomes. Ultimately, in the event of a planned surgery, perioperative GC stress dosing should be used as indicated per clinician discretion based on perioperative evaluation of cortisol status, regardless of whether or when osilodrostat is interrupted.

### Treatment Modifications for Pituitary Surgery

More specific scenarios were explored to recommend the best course of action for patients with CD undergoing pituitary surgery with an intent to cure (Table 1 and Fig. 3, statements 22 and 23). The consensus was to interrupt osilodrostat at least 5 days before surgery to ensure drug washout, given its half-life of approximately 4 hours [57], and to check preoperative serum cortisol levels for comparison with postoperative levels to facilitate the interpretation of routine postoperative cortisol measurements, which are generally used to gauge surgical remission. Of note, multiple advisors erred on the side of interrupting osilodrostat further in advance (ie, ≥7 days) to ensure adequate washout of any residual drug effects. Postoperative cortisol monitoring can be challenging to interpret if normal corticotrophs are not suppressed, which can happen with osilodrostat use. Therefore, even if cortisol levels do not significantly decrease in the immediate postoperative period, this may not necessarily mean absence of remission.

If a patient with CD is on concurrent spironolactone, the recommended best practice was to discontinue spironolactone after pituitary surgery until postoperative serum potassium levels are reassessed. While it may not be necessary for patients receiving low-dose spironolactone, discontinuation was considered prudent regardless of dose as spironolactone can affect aldosterone action for up to 6 weeks. However, continuing spironolactone may be warranted for patients with severe CD and persistent hypertension.

### Hypokalemia and Hirsutism Adverse Events

If patients develop hypokalemia on osilodrostat therapy, the consensus was to consider adding spironolactone or eplerenone to block cortisol and adrenal steroid precursor activation of mineralocorticoid receptors and/or add potassium supplements (Table 1 and Fig. 4, statement 24). Similarly, the US product label recommends considering adding mineralocorticoid receptor antagonists for patients with persistent hypokalemia despite potassium supplementation [23]. Spironolactone was preferred over eplerenone for patients with hypertension or for women with concomitant hirsutism. Spironolactone can also be considered for worsening or bothersome hirsutism in women, potassium levels and renal function permitting (Table 1 and Fig. 4, statement 25), although 1 advisor indicated that other antiandrogen therapies could also be considered. Importantly, women of child-bearing potential should also use an effective form of contraception while using spironolactone. Notably, patients using osilodrostat should also have magnesium levels monitored to correct hypomagnesemia if it develops [22, 23].

### Block-and-Replace Regimen for Special Populations

Cyclic CS is a rare subcategory of CS characterized by hypercortisolism “peaks” followed by physiological or hypocortisolemic cortisol “troughs” of indeterminate duration [58]. Advisors agreed that a block-and-replace regimen could be considered for patients who have intermittent symptoms and variable cortisol levels to decrease the risk of life-threatening AI during a trough (Table 1 and Fig. 4, statement 26). Patients with severe hypercortisolism (eg, UFC >5 × or >10 × ULN) may also benefit from a block-and-replace regimen with rapid escalation and higher osilodrostat dosages [45, 59, 60]. While there is no established consensus on a block-and-replace regimen, Bessiène et al demonstrated rapid titration of osilodrostat from 10 mg twice daily to 30 mg twice daily within 5 days, followed by block and replace beginning on day 5 with the highest dose of osilodrostat (total 60 mg daily), in a severely ill patient with ectopic CS [46]. The higher cost and potential side effects of block-and-replace therapy may be limiting factors when considering this therapeutic option.

### Statements That Did Not Achieve Consensus

Statements that did not achieve a consensus to include or exclude are summarized in Fig. 5. Though several biochemical tests were considered useful for different reasons, the panel had mixed opinions about whether such tests would be useful for monitoring treatment efficacy; rather, most advisors felt these tests were more appropriate for safety monitoring.

Most statements that did not achieve consensus were alternative actions proposed for a given clinical scenario, such that only 1 action per scenario was endorsed to represent the best course of action under most circumstances. The “alternative” options that did not achieve consensus still have relevance in some situations. For example, for a patient experiencing mild AI symptoms, 1 advisor would usually reduce osilodrostat dosage; however, they would maintain osilodrostat dosage with temporary GC treatment if the patient was also undergoing transient physical stress.

## Discussion

Consensus was achieved among experts from high-volume endocrinology centers regarding osilodrostat, a steroidogenesis inhibitor that was approved for CS in 2020 (EMA) and 2025 (FDA; expanded from prior CD indication in 2020) [22, 23]. Clinical recommendations are provided for osilodrostat initiation, baseline testing, initiating and managing dose changes, monitoring during dose titration, and modifying treatment for planned and unplanned clinical events, including any surgery for CS. Whereas consensus guidelines cover broad aspects of CS diagnosis, monitoring, and treatment [10], this study provides targeted practical guidance on optimal utilization of osilodrostat. These consensus statements are intended to serve as guidance for osilodrostat management across pituitary and nonpituitary ACTH-dependent CS and adrenal CS, with important caveats and exceptions noted for different clinical scenarios.

The consensus process found high agreement among experts on statements that were also largely consistent with the literature. Preparing patients with CS for success on osilodrostat was of high importance [29], and initiation dosage could be higher or lower than the label-recommended 2 mg BID depending on severity of CS and patient characteristics [43, 45, 46, 61]. Monitoring recommendations for efficacy and safety were largely consistent with the product label and general CS guidelines [10, 22, 23]. A single course of action was selected to represent the most broadly applicable option in different clinical scenarios. Universally applicable approaches were not always available because of the number of factors to be considered, including medical history, concurrent medications, and clinical characteristics. Generally, the Delphi panel would both interrupt osilodrostat and initiate GC stress dosing for symptoms and clinical findings suggestive of AI (including dizziness, vomiting, hypotension, postural vital signs, and hypoglycemia) and for unplanned surgeries. It is imperative that patients have adequate GC support during nonpituitary surgery regardless of whether or when osilodrostat is interrupted.

There were also areas in which consensus was not achieved, primarily related to laboratory testing and alternative acceptable approaches to clinical scenarios. Reasons cited for disagreeing with certain statements included that the statement lacked supportive evidence or depended on too many caveats. For statements related to testing, some advisors preferred to endorse only the most important or relevant tests.

Our study has several strengths. Achieving consensus using a modified Delphi process allowed advisors to score and comment on statements anonymously. Advisors with years of high-level experience managing patients with CS were represented, with at least 11 of the 13 advisors fully completing each step of the Delphi process. Statements cover a range of scenarios that a clinician could be expected to encounter in clinical practice. Of all the statement topics covered, only 2 statements (both in Survey 1) had a responder indicate a lack of experience with the topic. Though phase 3 clinical trial data with over 2 years of follow-up are available for osilodrostat [24, 25], further studies to address data gaps can be challenging because CS is a rare disease with multiple etiologies. Thus, consensus based on a collection of real-world experiences is of high value, particularly because this consensus study is specific to using osilodrostat. Statements supported by case studies and guidelines now hold the weight of approval from numerous experts, all of whom have experience with osilodrostat treatment, with additional years of experience beyond what has been covered in existing osilodrostat-inclusive guidelines.

Our study also has several limitations. The statements achieved clinical consensus rather than evidence-based consensus. Because a finite number of topics could be covered, data gaps remain for areas such as osilodrostat impact on interpreting inferior petrosal sinus sampling or postoperative ACTH/cortisol levels when assessing for postsurgical remission. All Delphi panel experts currently practice within the United States, where osilodrostat was only indicated for CD until April 2025. Though this entire Delphi consensus panel study was completed before the FDA approved the CS indication for osilodrostat, statements and discussions were inclusive of all etiologies of endogenous CS to reflect treatment indication in Europe and (now approved) use of osilodrostat for adrenal CS and ectopic ACTH syndromes in the United States.

## Conclusions

Clinical endocrinologists may not have sufficient opportunity to regularly use medications for rare diseases such as CS, which can be a barrier to prescribing new medications. Consensus statements among experts address the need for experience-based clinical guidance in the field and increase confidence for clinicians prescribing osilodrostat in specific clinical scenarios. Through a modified Delphi process, a panel of experts achieved consensus on 26 statements that provide guidance for clinicians interested in prescribing osilodrostat as medical therapy for CS. These recommendations are based on real-world clinical experiences from experts at high-volume clinics and provide valuable insight into the best practices for monitoring osilodrostat efficacy and safety and managing GWS/AI, intercurrent illness, and surgery, which will be invaluable for endocrinologists and all clinical providers who care for patients with CS.

## Data Availability

Survey scoring datasets that were generated and analyzed during the current study are available from the corresponding author upon reasonable request.
